# An optimized library for reference-based deconvolution of whole-blood biospecimens assayed using the Illumina HumanMethylationEPIC BeadArray

**DOI:** 10.1186/s13059-018-1448-7

**Published:** 2018-05-29

**Authors:** Lucas A. Salas, Devin C. Koestler, Rondi A. Butler, Helen M. Hansen, John K. Wiencke, Karl T. Kelsey, Brock C. Christensen

**Affiliations:** 1 Department of Epidemiology, Geisel School of Medicine, Dartmouth College, Lebanon, NH USA; 2 Department of Biostatistics, University of Kansas Medical Center, Kansas City, KS USA; 3 Departments of Epidemiology and Pathology and Laboratory Medicine, Brown University, Providence, RI USA; 4 Department of Neurological Surgery, Institute for Human Genetics, University of California San Francisco, San Francisco, CA USA; 5 Departments of Molecular and Systems Biology, and Community and Family Medicine, Geisel School of Medicine, Dartmouth College, Lebanon, NH USA

**Keywords:** DNA methylation, Epigenetics, Neutrophils, Monocytes, Natural killer cells, B-cells, Helper T-cells, Cytotoxic T-lymphocytes, Adults, Leukocytes

## Abstract

Genome-wide methylation arrays are powerful tools for assessing cell composition of complex mixtures. We compare three approaches to select reference libraries for deconvoluting neutrophil, monocyte, B-lymphocyte, natural killer, and CD4+ and CD8+ T-cell fractions based on blood-derived DNA methylation signatures assayed using the Illumina HumanMethylationEPIC array. The IDOL algorithm identifies a library of 450 CpGs, resulting in an average R^2^ = 99.2 across cell types when applied to EPIC methylation data collected on artificial mixtures constructed from the above cell types. Of the 450 CpGs, 69% are unique to EPIC. This library has the potential to reduce unintended technical differences across array platforms.

**Electronic supplementary material:**

The online version of this article (10.1186/s13059-018-1448-7) contains supplementary material, which is available to authorized users.

## Background

DNA methylation microarrays have become a widely utilized tool for epigenome-wide association studies (EWAS), including expanded use in studies investigating the association between DNA methylation with environmental exposures, and in the setting of case-control and longitudinal studies [1, 2]. Peripheral blood is the most commonly used biospecimen for these analyses primarily because it is easily accessible through a minimally invasive procedure, although some emerging evidence suggests that some specific DNA methylation changes in blood may reflect pathological states in target organs not easily or safely accessible by biopsy [3]. Finally, DNA methylation profiles in blood may, in some instances, summarize information of systemic exposures or diseases where cells from a single organ or tissue cannot be specifically assessed [4]. While some of the observed changes in DNA methylation reported in EWAS reflect induced epigenetic alterations within the constituent cells, others may reflect coordinately induced changes in the proportions of leukocyte subtypes in circulation that underlie or contribute to the pathophysiologic process. Both reference-based and non-reference-based techniques have been used to control the effect of cell heterogeneity, and thus possible confounding, in different studies, and their specific applications have been detailed elsewhere [5, 6]. Deconvolution techniques, such as constrained projection/quadratic programming (CP/QP) [7], provide a framework for estimating the relative proportions of blood cell types using blood-derived signatures of DNA methylation. So-called “deconvolution estimates” can then be used in downstream statistical models to adjust for the potential confounding effects of cell composition [4, 7–9], or examined independently to determine their association with risk or exposures [10–12]. Indeed, as in other general clinical applications, the ratio of myeloid to lymphoid lineages (neutrophil to lymphocyte ratio (NLR)) can be reconstructed in archival whole blood DNA samples measured with methylation arrays using different deconvolution approaches [11, 12]. Furthermore, while complete blood cell counts (CBC) are sometimes concurrently collected, deconvolution algorithms allow for estimation of lymphoid-specific subpopulations (e.g., B cells, CD4(+) T, CD8(+) T and natural killer (NK) lymphocytes), approximating the results of cell flow sorting without the need of additional sample input. This strategy helps to correct for inter-individual variation, lineage relationships (changes in the myeloid versus lymphoid ratio mean), and potential effects of prominent methylation differences in general cell subpopulations.

Current analysis pipelines for estimating the proportions of leukocyte subtypes in adult blood are based on samples from six adult males that were purified with flow cytometry and profiled for DNA methylation using the Illumina HumanMethylation450K platform (450 K array) [13]. As the 450 K array is now the predecessor to the recently released Illumina HumanMethylationEPIC array (EPIC array), an outstanding question relates to accuracy of cell deconvolution of peripheral blood measured on the EPIC array using existing 450 K reference methylation signatures. The EPIC array interrogates > 860,000 CpG sites, roughly twice the number of CpGs contained on the 450 K array, with additional genomic content in enhancer regions and DNase hypersensitive sites (DHS), which are important in hematopoietic development and differentiation [14, 15]. In this work, we extend the available reference library for deconvolution of blood cell proportions using the EPIC array with the goal of both improving the accuracy of cell composition estimates and overcoming potential technical differences in platforms [16, 17]. Using antibody bead sorted neutrophils, B cells, monocytes, NK cells, CD4+ T cells, and CD8+ T cells, we measured DNA methylation with the 850 K EPIC DNA methylation array and applied an iterative algorithm for Identifying Optimal Libraries (IDOL) from leukocyte differentially methylated regions (L-DMR) that improves the accuracy and efficiency of cell composition estimates obtained by cell mixture deconvolution [18]. We then compared the performance of cell estimates obtained using the EPIC platform and optimized library to the now unavailable 450 K array in artificial blood mixtures with predefined cell proportions.

## Results

The FlowSorted.Blood.EPIC dataset contains information from neutrophils (Neu, *n* = 6), monocytes (Mono, *n* = 6), B lymphocytes (Bcells, n = 6), CD4+ T cells (CD4T, n = 7, six samples and one technical replicate), CD8+ T cells (CD8T, n = 6), natural killer cells (NK, n = 6), and 12 DNA artificial mixtures (labeled as MIX in the dataset). Across samples, the average purity reported in the control flow sorting (after antibody-linked magnetic bead sorting) was 95% (range 88 to 99%), with Mono having the lowest purity across the six cell subtypes (Additional file 1: Figure S1). Individual sample cell purity is reported in the phenotype table in the FlowSorted.Blood.EPIC file. We explored potential genetic clustering using the ethnicity and the 59 control SNP probes included in the array. A striking clustering was observed when grouping for larger ethnic groups (Additional file 1: Figure S2). Using a principal component regression analysis, the first 20 principal components were tested against potential confounders (Additional file 1: Figure S3). Each of the first five principal components were significantly (*P* < 0.01) associated with cell type composition. Other potential confounders, including body mass index (BMI; *P* < 0.01), subject weight (*P* < 0.01), age (in years; *P* < 0.01) or sex (*P* < 1 E-04) were only accounted for in the sixth to eleventh principal components, and smoking was significant only with the 12th component (*P* < 0.05). Cell purity was associated with the ninth principal component (*P* < 0.05). In contrast to what was observed using the genetic information, ethnicity was not significantly associated with any of the top 20 principal components.

**Fig. 1:**
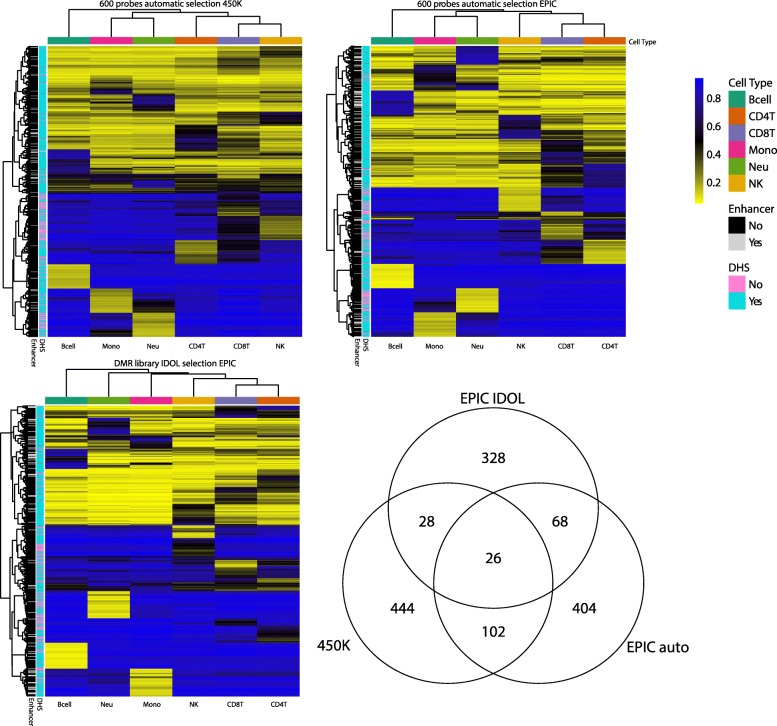
Comparison of L-DMR libraries among automatic selection in minfi and the IDOL algorithm for optimization. **a** Reinius reference dataset [13] probes from the 450 K array (n = 600 CpGs). **b** Probes selected from the new reference samples measured with the EPIC array (*n* = 600 CpGs). **c** L-DMR library derived from IDOL using the EPIC array (*n* = 450 CpGs). **d** Overlapping of the probes of the three methods. *DHS* DNase hypersensitive sites

We used three deconvolution methods for comparison (see “Methods” for details): 1) the current commonly used 450 K reference (Reinius et al.) [13] using the automatic selection to identify the L-DMR library in the minfi Bioconductor package [19]; 2) our new EPIC reference using the automatic selection to identify the L-DMR library in minfi; 3) our new EPIC reference using IDOL selection to identify the L-DMR library [18]. The automatic selection picks the top 50 hyper- and hypomethylated probes for each cell type (600 probes total), while the IDOL method identified 450 probes as the optimal number of probes for deconvolution (Additional file 1: Figure S4). The probes selected by the various methods are compared in Fig. 1. Only 26 probes overlapped across the different methods. A comparison of the selected probes, including the proportion per genomic context and probes in Phantom5 enhancers and DHS, is provided in Table 1. The majority of the probes selected for deconvolution using the new EPIC array were not present on the previous 450 K array; 66% of the probes selected using the automatic selection method and 69% of the probes selected with IDOL were unique to the EPIC array. As expected, more probes in the open sea were selected using the new reference library (80 and 76% using the automatic and IDOL methods, respectively, compared to 57% using the 450 K reference). In addition, approximately twice as many Phantom5 enhancer sites were selected using the new EPIC platform with the automatic (18%) and EPIC methods (16%) compared with the Reinius [13] reference probe set from the 450 K platform (9%).

**Table 1:** Genomic context of CpG sites selected for each L-DMR library approach

	Automatic selection 450 K	Automatic selection EPIC	IDOL EPIC	*P* ^a^
Probes	(*n* = 600)	(*n* = 600)	(*n* = 450)	
	N(%)	N(%)	N(%)	
CpG present on 450 K array	600 (100)	203 (34)	140 (31)	7.50E-29
Enhancers (Phantom5)	53 (9)	108 (18)	70 (16)	3.13E-69
DNase hypersensitive sites	452 (75)	467 (78)	328 (73)	6.66E-147
Genomic context				
CpG island	47 (8)	24 (4)	10 (2)	4.35E-62
Shores	116 (19)	55 (9)	63 (14)	
Shelves	95 (16)	41 (7)	35 (8)	
Open sea	342 (57)	480 (80)	342 (76)	
Functional context				
TSS1500	73 (12)	44 (7)	38 (8)	2.10E-102
TSS200	46 (8)	23 (4)	11 (2)	
5′ UTR	76 (13)	69 (12)	46 (10)	
First exon	20 (3)	16 (3)	7 (2)	
Body	241 (40)	283 (47)	210 (47)	
3′ UTR	29 (5)	11 (2)	9 (2)	
Intergenic	115 (19)	152 (25)	128 (28)	

^a^*P* is calculated from the χ^2^ test comparing the proportions between the three L-DMR selection methods

**Fig. 2:**
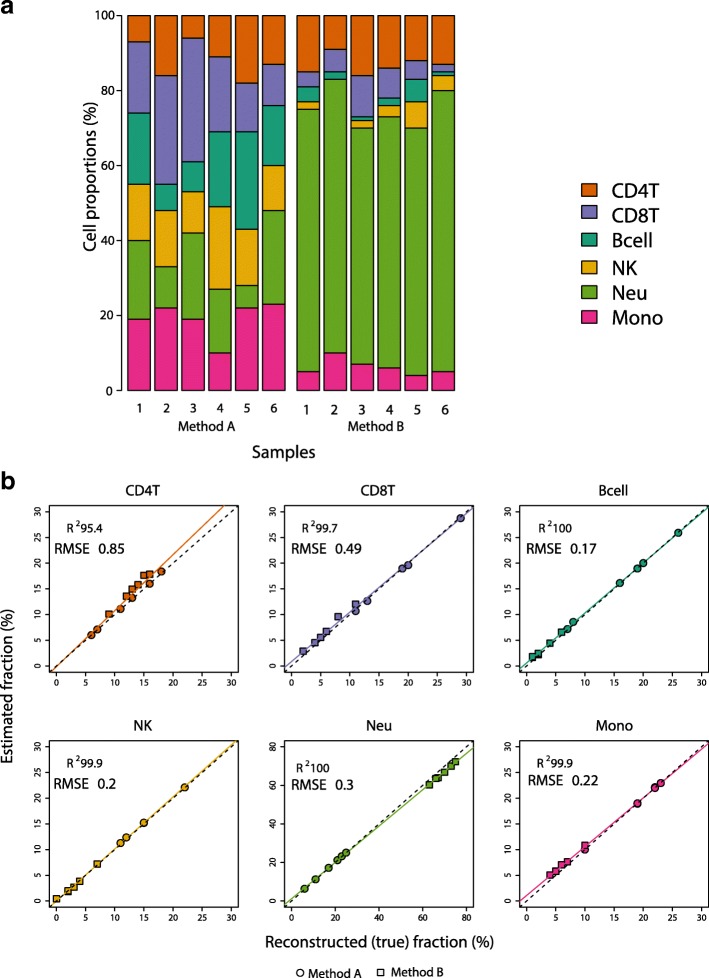
Comparison of estimate cell proportions using constrained projection/quadratic programming (CP/QP) versus the reconstructed (true) DNA fraction in the artificial DNA mixtures using the EPIC IDOL method. **a** Cell-specific DNA proportions per sample included in the two mixture reconstruction methods (methods A and B). **b** R^2^ and RMSE using the EPIC IDOL method and the two reconstruction methods

**Fig. 3:**
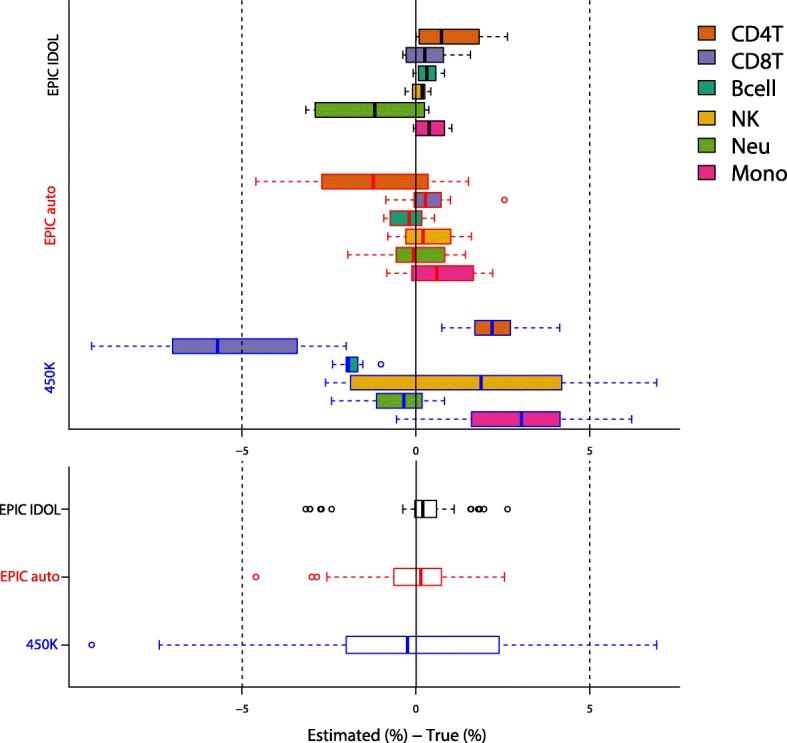
Observed estimates of absolute error by deconvolution method per cell type (*top panel*) and global per method (*bottom panel*)

Once we determined the probes for cell type estimation, we used the minfi modified Houseman constrained projection approach [7] to estimate the cell composition of 12 samples, spread across two sets of artificially reconstructed mixtures. As the specific amount of DNA per cell type in each mixture was known, we compared our estimate of cell proportions to the amount of DNA represented by that cell type in each of the artificial mixtures (Fig. 2a, Additional file 1: Table S1). The R^2^ (coefficient of determination) values were > 86% across all cell types and across the three tested methods (Additional file 1: Figure S5). However, we consistently obtained better cell type proportion estimates (higher R^2^ and lower RMSE (root mean square error)) when using the L-DMR library generated with the IDOL method from EPIC platform methylation data, and the variance of our estimates was consistently lower (Fig. 2b, Additional file 1: Figure S5). For all the cell types, except CD4T, the R^2^ was over 99.7%. The lowest R^2^ estimate from applying the IDOL method to the EPIC platform data was for CD4T (R^2^ = 95.5%). The observed versus expected estimate for CD4T was slightly better when using the 450 K L-DMR library (R^2^ = 98.1%), and performance was worse using automatic selection with data from the EPIC platform (R^2^ = 86.0%). Although the results are highly correlated to the actual proportion of DNA in the artificial mixtures when using the Reinius [13] 450 K reference L-DMR library, the estimates showed increased variability compared to estimates obtained using the EPIC reference L-DMR library (Fig. 3). Importantly, the magnitude of the variance was strongly significantly lower using IDOL compared to companion automatic methods (Bartlett test *P* = 7.60 E-26).

As a sensitivity analysis, we compared the results of the IDOL library using CP/QP (minfi method) versus two additional deconvolution methods: 1) CBS-CIBERSORT, a support vector machine non-constrained projection; and 2) and Robust Partial Correlation (RPC) a linear non-constrained projection using the methods described by Teschendorff et al. and available in the R package EpiDISH [14]. Using a paired *t*-test, we compared the true values (fraction of cell DNA in the artificial mixture) versus the estimates obtained by the three methods; the global mean differences for all the cells were not statistically significant (*P* > 0.05 for the three tests). The paired mean differences were analyzed using Bland-Altman plots (Additional file 1: Figure S6). Subtle, less than 2%, mean differences were observed in the cell by cell estimate comparisons across each of the deconvolution methods. Across the three deconvolution methods, there were no statistically significant differences between the true values for CD8T cells and their estimated fraction (*P* > 0.05). RPC mean estimates were closer to the true values for CD4T, though CBS underestimated CD4T (mean difference = − 0.8%), and CP/QP overestimated CD4T (mean difference = 1.0%). RPC estimates were also closer to the true Bcell values, whereas both CBS and CP/QP overestimated this cell type (mean differences = 0.6 and 0.4%, respectively). NK cells were slightly underestimated using CBS (mean difference = − 0.3). All three methods overestimated monocytes by a small percentage that was statistically significant (*P* < 0.05). The lowest mean difference in monocytes was observed with CP/QP (0.5%), followed by RPC (0.7%) and CBS (1.6%). CBS outperformed RPC and CP/QP for neutrophil estimation (*P* > 0.1), and both CP/QP and RPC underestimated neutrophils (mean differences = − 1.27 and − 1.66%, respectively).

### Pathways in the selected EPIC IDOL library

The probes present in the new EPIC IDOL L-DMR library were tested for enrichment using missMethyl and the Gene Ontology (GO) and the Gene Set Enrichment Analysis (GSEA) set 7 (immune related) v.6.1 pathways. In total, 375 GO pathways (299 biological processes, 31 cell components, and 45 molecular features) and 181 GSEA set 7 pathways were statistically significant (false discovery rate < 0.05) after array bias correction (Additional files 2 and 3). Among others, several GO pathways were tracked to the parent GO terms response to wounding (e.g., inflammatory response, defense response), T-cell activation (e.g., T-cell activation) and leukocyte proliferation. The GSEA pathways included pathways related to the different six cell types and other cells derived from the six cells included in the database.

### Potential applications

**Fig. 4:**
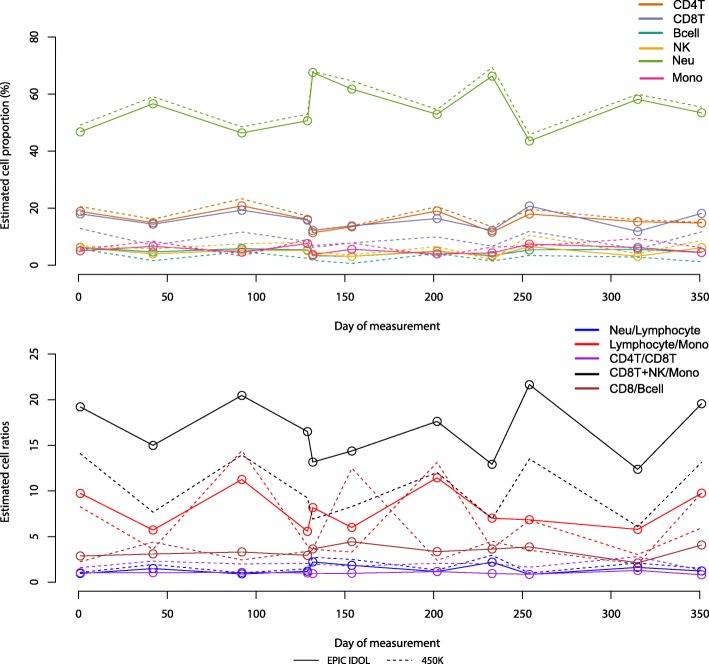
Comparison of the longitudinal assessment of cell type proportions and cell ratio changes using DNA methylation data and two different reference L-DMR libraries (EPIC IDOL and 450 K)

Although any EWAS using the IlluminaHumanMethylationEPIC array could benefit from estimates derived from the new reference library, one specific setting in which the use of more precise cell estimates is particularly beneficial includes longitudinal and/or repeated measurement studies. Using a dataset containing peripheral blood DNA methylation information from one volunteer, 11 repeated measurements were obtained across 350 days of observation. Although the measurements were from a healthy male adult volunteer, we observed an important range of variability in the cell subpopulations across the different time points (Fig. 4). Specifically, we observed a potential underestimation of CD8T (− 5.5% in the 450 K estimates, *P* = 2.86E-05) and Bcell (− 3.84% in the 450 K estimates, *P* = 1.10E-04). Further, when examining the cell ratios (lineage relationships) those ratios containing these cell subpopulations, and in particular those including CD8T cells alone, were dramatically affected (CD4T/CD8T increased 5.82 points, *P* = 5.76E-05; CD8T + Mono/NK were reduced 4.78 points, *P* = 1.28E-04). In the CD8T/Bcell ratio, although the mean change was non-statistically significant, the global variance of the ratio was affected (Bartlett test *P* = 4.64E-05). Importantly, the neutrophil estimates were preserved using any of the reference datasets, though the neutrophil to lymphocyte ratio (NLR) estimates were slightly higher using the 450 K compared to the EPIC IDOL L-DMR library (1.97 points, *P* = 0.06). Subtle changes are better captured using the new L-DMR library. In addition, the discordance in the CD8T and Bcell estimates using the 450 K L-DMR library compared with the EPIC array library (underestimation in the reconstructed samples comparisons) was consistent with the direction and magnitude in the error observed when we compared the performance with the reconstructed samples (Fig. 3).

To interrogate deconvolution using datasets from the previous Illumina HumanMethylation450K platform, we optimized a second set IDOL L-DMR library from our data, measured on the EPIC platform, but including only probes also present on the 450 K array. Restricting to CpGs also on the 450 K array, a set of 350 probes was identified as the optimal IDOL L-DMR library (similar R^2^ and lower RMSE than libraries containing less or more probes), 60 of which are part of our IDOL EPIC L-DMR library. The performance of the 450 K-restricted 350 CpG L-DMR library in 12 artificial reconstructed mixtures (GSE77797) measured with the 450 K platform (Additional file 1: Figure S7) was consistent with the performance of the 450 CpG L-DMR library in the EPIC samples (Fig. 2). In particular, slightly higher RMSEs and slightly lower R^2^ values were observed (Additional file 1: Figure S7); however, the RMSEs were lower and the R^2^ values were higher, or similar, to those reported previously by Koestler et al. [18] using the Reinius reference [13].

**Fig. 5:**
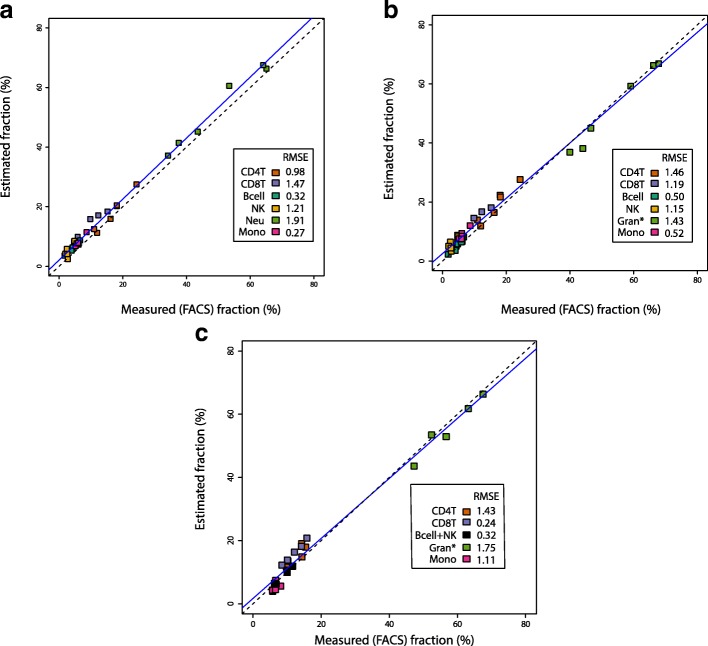
Comparison of the estimated cell proportions using constrained projection/quadratic programming (CP/QP) versus the FACS measured fraction in EPIC and 450 K platforms. **a** Whole blood cell samples arrayed using the EPIC platform with known (FACS) fractions for the six main cell subtypes. Cell estimates were obtained using the EPIC IDOL method. **b** Whole blood cell samples arrayed using the Illumina 450 K platform with known (FACS) fractions for the six main cell subtypes. Cell estimates were obtained using the EPIC IDOL 450 K legacy method. **c** Five out of 11 observations on the longitudinal dataset run with EPIC had FACS information

Finally, to further validate our estimation using actual samples, we estimated the cell composition of whole blood samples collected from six additional healthy donors, whose DNA was run on the EPIC platform, and compared our estimates to FACS measured cell proportions collected on the same samples (GSE112618; Fig. 5a). We also deconvoluted six publicly available samples with available FACS information arrayed using the 450 K platform (GSE77797; Fig. 5b), and in five of the 11 samples in our longitudinal dataset with FACS information at the time of the sample collection (years 2011 and 2012), we compared our estimates of cell composition in archival DNA samples arrayed using the EPIC platform to the corresponding FACS measured proportions (GSE110530; Fig. 5c). In all three datasets, RMSEs were less than 2.0 for all cell types (Fig. 5). The least accurate estimations were observed in the granulocyte/neutrophil fractions, where increased accuracy was observed when comparing the total granulocytes vs neutrophil estimates compared to the total neutrophils vs neutrophil estimates. In particular, in Fig. 5a, the RMSE for neutrophils was 1.91, whereas when we instead used total granulocytes (the sum of neutrophils, eosinophils, and basophils), the RMSE was reduced to 0.92.

## Discussion

Here, we offer a new DNA methylation reference library from the Illumina EPIC array for six adult blood cell subtypes. Using artificial mixtures with fixed proportions of purified cell DNA, this library offers more precise results in terms of the cell estimations obtained through constrained projection compared to those using the previous 450 K reference library. Although the statistical differences are subtle numerically, the global increased precision may help to control the confounding of cell subpopulations in studies using adult peripheral blood.

In our approach, we suggest that optimized probe selection using IDOL may also help to increase precision and reduce noise compared to larger probe lists based solely on *t*-statistic ranking of cell-specific hyper- and hypomethylated CpGs. Nevertheless, even when using the more extended automatic selection approach, the global results are similar, albeit less precise. Furthermore, although the results were statistically similar using the previous 450 K reference library, our results suggest that this L-DMR library was less precise in their estimations.

**Fig. 6:**
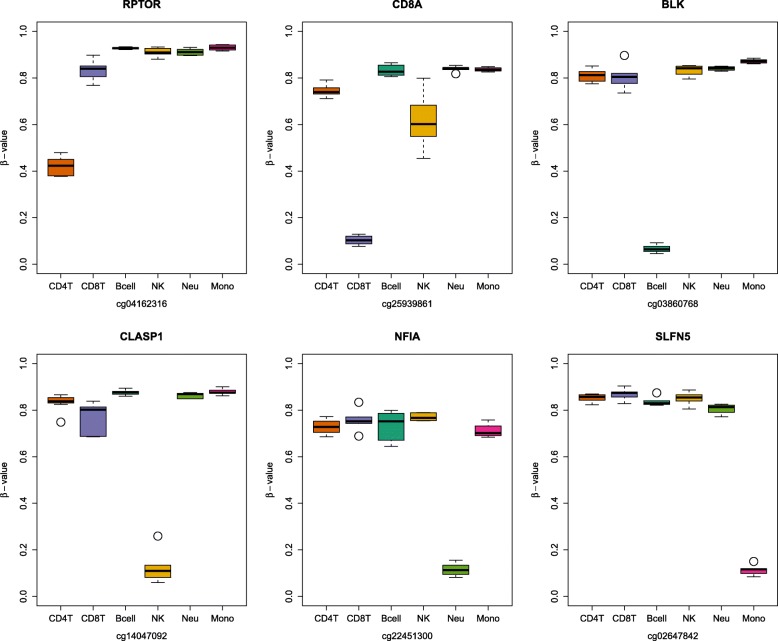
Examples of critical CpGs for cell deconvolution selected by IDOL

The increased coverage of the new EPIC array may also include additional important genomic areas for hematopoiesis and immune cell development. Although IDOL relies on an algorithmic approach for the selection of probes, the output L-DMR library contains probes associated with critical biological pathways in immune development and differentiation. Through its iterative selection of CpG loci that optimize prediction performance across the six leukocyte subtypes, the IDOL algorithm identifies critically sensitive and specific differentially methylated sites that populate the final deconvolution library. Included among the cell-specific output are loci that figure prominently in established leukocyte biology, as well as others that are less well described. Examples of the former are loci that reside in genes presented in Fig. 6. *BLK* (B lymphoid tyrosine kinase) is well established in B-cell antigen receptor signaling and B-cell development [20]. *CD8A* (CD8 alpha subunit) is a cell defining co-receptor for cytotoxic T-cell receptor–MHC–antigen complex response [21]. Although this molecule is also expressed in approximately 40% of NK cells [22], the use of this marker in conjunction with other probes help to differentiate this specific cell type. *NFIA* (nuclear factor 1 A transcription factor) in concert with *miR-223* is a crucial player in the molecular circuitry controlling human granulopoiesis [23, 24]. IDOL also selected loci in the *RPTOR* gene (regulatory associated protein of MTOR) to discriminate CD4T cells. Metabolic reprogramming mediated by *RPTOR* is emerging as essential in T helper cell differentiation [25]. In previous studies, a different set of CpGs related to RPTOR have been associated with inflammatory markers in CD4T [26]. Interestingly, *SLFN5* (Schlafen factor 5 protein) demethylation strongly delineates monocytes from neutrophils and other cell types. Schlafen family proteins are alpha interferon-inducible growth and cell cycle regulatory proteins but have no known function in monocyte biology [27]. Finally, a notable NK-specific locus was uncovered within the *CLASP1* gene (cytoplasmic linker associated protein 1). CLASP proteins are important in microtubule organization and vesical transport [28] but a role for this gene in NK biology has not been described. Further experimentation is required to elucidate the mechanistic connections between specific DNA methylation events and the functional characteristics of diverse immune cells. The present results demonstrate notable improvements in understanding the contributions of immune cell compartments to the substantial variation in DNA methylation observed in peripheral human blood.

In non-pathological conditions, less common cell subpopulations will probably be estimated as part of the closest cell in the cell development hierarchy (as observed with the estimates of the neutrophils approximating the total granulocytes in our FACS comparisons). However, a limitation of the current approach is its potential vulnerability in pathologic conditions wherein other cell types or cell transition states may appear in the peripheral blood. The accuracy of reference-based cell deconvolution approaches can potentially be affected by the presence of cell populations that are unaccounted for in cell reference libraries. One example is the presence of nucleated red blood cells in cord blood samples; this heterogeneous group of erythroid cells shows a characteristic unmethylated pattern, previous to enucleation [29], that could affect the estimates in that specific age group where they are relatively abundant (about 5% of the nucleated cells) but disappear in the first 72 h after birth [30–32]. However, in normal conditions, independent of the age of the subject, it is expected that cell types or states without a direct reference methylome will be accounted for as part of the closest cell subtype with a reference. In fact, in our data we observed how the FACS information confirmed that most of the variability in a longitudinal assessment of a healthy subject was attributable to changes in the cell population proportions. The approach described here can accommodate additional normal or pathologically related cell types, but as with all deconvolution methods additional cell libraries and optimization procedures would need to be applied to identify and minimize estimation bias arising from an additional cell population. These limitations are not unique to epigenetic approaches as conventional FACS itself requires prior knowledge of cell characteristics for accurate cell profiles.

This new reference library has the potential to be widely used in the newest adult peripheral blood EWAS. The use of an EPIC-specific reference library will eliminate unintended technical differences arising from applying a reference library from a previous generation array which may result in residual confounding or critical technical defects which go beyond the cell heterogeneity problem when analyzing blood samples. The use of reliable cell reference panels is particularly important for longitudinal assessment of cohort datasets. Using information from the 450 K datasets, it had been shown that temporal trends in longitudinal studies were mainly driven by changes in cell composition across time [33–36]. The expected increase in precision using the new reference would be especially important in this context to control for aging-related effects changing specific subpopulations of T lymphocytes in which a higher variability is expected when using the previous library. Indeed, this library may also find particular utility when additional subtypes of leukocytes are added to the current library, as is evidenced by the plethora of EPIC-array-specific L-DMR loci discovered in the new analysis.

## Conclusions

This new EPIC-specific reference library will reduce residual confounding arising from the use of a reference library from a previous array generation when analyzing adult blood samples. The increased precision of using this new L-DMR library will help in applications where subtle changes in specific cell subpopulations may lead to higher than expected variability, such as longitudinal studies.

## Methods

In this work, we extend the available reference library for deconvolution of blood cell proportions using the EPIC array with the goal of both improving the accuracy of cell composition estimates and overcoming potential technical differences in platforms. Using magnetic sorted neutrophils, B cells, monocytes, NK cells, CD4+ T cells, and CD8+ T cells, we measured DNA methylation with the 850 K EPIC DNA methylation array and applied IDOL to identify optimal L-DMR libraries. We then compared the performance of cell estimates obtained using the EPIC platform and optimized the L-DMR IDOL library to the now unavailable 450 K array in artificial blood mixtures with predefined cell proportions.

Six MACS-isolated and FACS-verified purity cell subtypes (neutrophils (Neu), monocytes (Mono), B lymphocytes (Bcells), T helper lymphocytes (CD4T), T cytotoxic lymphocytes (CD8T), and natural killer lymphocytes (NK)) were purchased from AllCells® corporation (Alameda, CA, USA) and STEMCELL technologies (Vancouver, BC, Canada). Cells were isolated from 31 males and 6 females, all anonymous healthy donors. The donors had a mean age of 32.6 years (range 19–59 years) and an average weight of 86 kg (range 65–118 Kg) and were negative for HIV, HBV, and HBC. Women were not pregnant at the time of sample collection, and samples were collected from donors with no history of heart, lung, or kidney disease, asthma, blood disorders, autoimmune disorders, cancer, or diabetes. All donors provided written informed consent before donation. The full phenotype information is available in the FlowSorted.Blood.EPIC package [37] and in the Gene Expression Omnibus (GEO; GSE110554) [38].

Isolation protocols are available through the commercial websites of AllCells and STEMCELL technologies. In brief, cells were selected using immunomagnetic labeling through two different protocols: 1) for Neu, leukocytes were separated using HetaSep followed by density gradient separation and neutrophil negative selection; 2) Mono, Bcells, NK, CD4T, and CD8T were negatively isolated from untouched peripheral mononuclear cells using indirect immunomagnetic cell labeling systems (CD14, CD19, CD56, CD4T, and CD8T, respectively).

Twelve artificial mixtures were reconstructed using DNA from the specific cell samples. Two different sets of reconstruction mixtures, each with *n* = 6, were determined by randomly generating proportions from a six-component Dirichlet distribution. The first set of reconstructed samples (method A samples) used mixtures of purified leukocyte subtype DNA in relatively equivalent proportions across the six leukocyte subtypes. For the second set of six samples, the proportions of DNA for each leukocyte subtype were selected to resemble their relative fractions in the peripheral blood of normal human adult subjects (method B samples). A mixture containing 1.2 μg of total DNA was estimated using the proportions included in Additional file 1: Table S1. The DNA from the cell sorted samples and those of the artificial mixtures were randomized in the slide slots of the microarray. Sample DNA (1 μg) was bisulfite converted and processed according to the Illumina protocols at the Vincent J. Coates Genomics Sequencing Laboratory at UC Berkeley.

The raw idat files from the EPIC methylation array were pre-processed using minfi [19] and EnMIX [39] for quality control using R v.3.4.3 [40]. To assess data quality, we used a detection *P* value of 1E-06, three standard deviations of the mean bisulfite conversion control probe fluorescence signal intensity, and a minimum of three beads per probe. Only 1897 CpGs had a detection *P* > 1E-06 in 5% or more of the samples; however, they were not masked in the raw dataset. No samples were excluded because of low quality. The IDOL EPIC L-DMR library is freely available in Bioconductor as the package FlowSorted.Blood.EPIC [37] to be adopted in downstream analyses in current analyses pipelines. The package contains a RGChannelSet R object generated through minfi containing 49 samples and information on 1,051,815 probes corresponding to 866,091 CpGs using the latest annotation release by Illumina (MethylationEPIC_v-1-0_B4). It is important for the reader to note that the cells were purified using an immunomagnetic procedure; the name “FlowSorted” was kept for easy adoption and integration with previous minfi pipelines.

### IDOL algorithm

For a complete description of the IDOL algorithm please refer to Koestler et al. [18]. In brief, the IDOL algorithm utilizes a training dataset consisting of both blood-derived DNA methylation data and measurements of the fraction of each of the underlying cell types (e.g., FACs, artificial mixtures of DNA from purified cell types of pre-specified, known proportions, etc.) as a means to identify optimal reference libraries for cell mixture deconvolution. A series of *t*-tests comparing the mean CpG-specific methylation between each leukocyte cell type compared to the mean methylation across all the other cell types was conducted to identify discriminating CpGs (e.g., L-DMRs) for each specific cell type. Based on this analysis, CpGs were then rank-ordered on the basis of their *t*-statistics and the *L*/2 CpGs with the largest and smallest *t*-statistic for each K cell type were identified and pooled. *L* is a tuning parameter representing the number of cell-specific L-DMRs and was set to *L* = 150 in our application, consistent with Koestler et al. [18]. A candidate L-DMR library containing the total L*K unique L-DMRs for each cell type forms the search space for the IDOL algorithm, from which L-DMR subsets of size < L*K are sequentially and probabilistically selected and examined for their prediction accuracy in deconvoluting the samples in the training dataset. The user needs to preselect the library size in order to balance accuracy and precision of cell composition estimates. For the application of IDOL presented here, we considered libraries ranging from 50 to 800 CpGs by increments of 50, as our previous work has shown that libraries ranging from 300 to 600 CpGs generally yield accurate and reliable deconvolution estimates. In the first iteration of the IDOL algorithm, all L*K CpGs constituting the candidate library have an equal probability of being selected to be included in the DMR subset library. Using the randomly assembled DMR library, the constrained projection/quadratic programming approach [7] is applied to obtain cell composition estimates for each sample in the training dataset. Using these predictions, the R^2^ and RMSE (root mean square error) were calculated for each of the cell types (Additional file 1: Figure S4), comparing the cell estimates to their known proportion in each sample. One-by-one CpGs are removed from the randomly selected DMR library, followed by computation of R^2^ and RMSE based on cell composition estimates obtained using a library consisting of only the remaining CpGs. This procedure allows assessment of the contribution of each CpG in the library in terms of its impact on the accuracy of cell composition estimates and, in doing so, provides a basis for modifying the probability of each CpG being selected in subsequent IDOL iterations. This process is repeated at each iteration, with the algorithm eventually converging on an “optimal” library for deconvolution. Per Koestler et al. [18], we used 500 iterations in our implementation of IDOL.

### Deconvolution methods

We used three different deconvolution methods to assess the performance of the new reference library. First, we used the estimateCellCounts function contained in the minfi Bioconductor package [19, 41]. estimateCellCounts is an adaptation of the Houseman et al. CP/QP method [7], in which a raw reference library is combined and normalized with a target dataset, followed by cell deconvolution. By default, this method uses the FlowSorted.Blood.450 K library derived from the Reinius dataset [13] as the reference dataset. Both the reference and target datasets are normalized together using independent type I and type II probe quantile normalization [42]. First, the default library used for cell mixture deconvolution consists of 600 CpGs, representing the top 50 hyper- and hypomethylated CpGs, rank-ordered based on the *t*-statistic obtained in comparisons of CpG-specific methylation between each cell type (i.e., CD4T, CD8T, NK, Bcell, Mono, and Neu) and all other cell types. We hereafter refer to this approach as automatic selection 450 K. Second, we used the same estimateCellCounts defaults but substituted FlowSorted.Blood.450 K with FlowSorted.Blood.EPIC as the underlying reference dataset. Similar to the previous approach, the top 50 hyper- and hypomethylated CpGs were identified for each cell type and used to assemble the library for deconvolution consisting of 600 total CpGs. We hereafter refer to this approach as automatic selection EPIC. Finally, we used IDOL for probe selection [18]. This approach dynamically scans a candidate set of cell-specific methylation markers to find libraries that optimize the accuracy of cell fraction estimates obtained from cell mixture deconvolution. Library sizes ranging from 50 to 800 CpGs, in increments of 50, were considered (see IDOL algorithm above for details). The selected probes (*n* = 450, IDOL optimized L-DMR library), plus the genomic context information, are supplied as Additional file 4. Per each cell type the following number of probes were selected: Bcell 71, CD4T 70, CD8T 82, Mono 72, Neu 73, and NK 82. As both the reference and the target were EPIC datasets, we changed the default normalization and only used the methylumi-noob background correction [41] before the cell projection. This last method is referred to as IDOL selection EPIC. As this method is not included within the estimateCellCounts function, we offer a modified function in our package named estimateCellCounts2 which allows all the options already included in the original function plus the use of IDOL-customized probe selection. The estimates of the three methods were compared against the proportion of cell DNA included in the mixture (true value); we report the R^2^ and the RMSE (residual mean standard error) for the three methods. The absolute mean error was calculated subtracting the estimated proportions from the reconstructed (true) fraction spiked in the sample. As a measure of the global variance variability we used a Bartlett test for homogeneity of the variances to compare the three methods. As a sensitivity analysis we compared the results of the EPIC IDOL library using CP/QP (minfi method) versus two additional deconvolution methods: 1) CBS-CIBERSORT, a support vector machine non-constrained projection; and 2) RPC (robust partial correlation), a linear non-constrained projection using the methods described by Teschendorff et al. and available in the R package EpiDISH [14]. We used a paired *t*-test to compare the true values (fraction of cell DNA in the artificial mixture) versus the estimates obtained by the three deconvolution methods.

### Longitudinal dataset

A repeated measurement dataset (GSE110530) [43] was from a male adult volunteer who provided 12 samples of blood distributed over a period of 350 days from March 2011 to February 2012. The DNA was extracted from whole blood within 24 h of sampling and archived at − 80 °C. Total input DNA of 0.75 μg, as measured by Quant-iT Picogreen dsDNA Assay (Invitrogen, Carlsbad, CA), was prepared for each time point. Samples were randomized across the slots of the microarray. Bisulfite conversion and processing were performed according to Illumina protocols using the IlluminaHumanMethylationEPIC array at the Vincent J. Coates Genomics Sequencing Laboratory at UC Berkeley. During quality control, one of the samples (time point 11) showed a different SNP content pointing to a potential sample mix-up and was excluded from this analysis. We estimated the cell composition using the 450 K L-DMR and the EPIC IDOL L-DMR methods and compared the mean difference and homogeneity of the variance of the estimates between both methods using *t*-test and the Bartlett test.

### Extension to whole blood samples and application for legacy 450 K datasets

As a potential extension and validation of our algorithm, we used a public dataset (GSE77797) [44] containing 12 samples of artificial mixtures and six whole blood samples with known flow-sorted fractions for the main six cell fractions arrayed using the Illumina HumanMethylation450k platform. We optimized an EPIC IDOL 450 K legacy L-DMR library using the same procedure described for the EPIC IDOL L-DMR above. The resulting library contained 350 probes present on the previous 450 K Illumina DNA methylation array generation (Additional file 5). The estimated cell composition using this EPIC IDOL 450 K legacy L-DMR library was compared against the reconstructed fraction or the FACS measured fraction. We report the R^2^ and the RMSE for the artificial mixtures and the RMSE for the FACS measured samples.

### Validation using samples with FACS information

Three independent datasets were used for validation. We ran six samples of healthy donors with FACS information using the EPIC platform (GSE112618) [45]. FACS analyses were carried out on whole blood provided by six healthy blood donors using established methods as described in Accomando et al. [46]. In brief, the gating strategy included counting total leukocytes using CD45(+), granulocytes based on CD16 and CD15, monocytes based on CD14, total T lymphocytes marked as CD3(+), CD4T were CD3(+) CD4(+), CD8T were CD3(+) CD8(+), B lymphocytes marked as CD19(+), and NK as CD56(+). We also compared the performance of our estimations against six additional samples with FACS information available in GEO which were arrayed using the Illumina HumanMethylation450k array (GSE77797) [44]. Finally, for five of the 11 samples of our longitudinal dataset analyzed with the EPIC platform, we had partial FACS information. In this latter dataset we show the CD3(−) lymphocyte fraction as the sum of Bcell and NK. DNA isolated from donor blood was stored at − 80 °C for approximately 6 years before being assayed on the EPIC array. Data are available in the GEO (GSE110530) [43].

### Test for enrichment

The IDOL L-DMR library was tested for enrichment using the GO database version 3.5.0 with date 11/08/2017, and the immune curated GSEA (set 7) version 6.1 using missMethyl to correct for array bias [47]. Only those pathways containing more than ten probes of the L-DMR library and pathways with less than 2000 genes were selected for this analysis. Pathways with a false discovery rate < 0.05 were considered statistically significant.

## Additional files

Additional file 1: **Figure S1.** Estimated cell purity by flow cytometry per cell type. **Figure S2.** Heatmap based on a hierarchical cluster of purified cell types and cell mixtures based on the array SNPs. **Figure S3.** Association between the top 20 principal components and potential confounders for DNA methylation. **Figure S4.** Iterative testing of different L-DMR library sizes using the IDOL optimization algorithm. **Table S1.** Cell composition percentages for the artificial reconstruction samples. **Figure S5.** Comparison of several probe selection methods and estimated cell proportions using constrained projection/quadratic programming (CP/QP) versus the reconstructed (true) DNA fraction in the artificial DNA mixtures. **Figure S6.** Bland-Altman plots comparing the mean differences between the estimated cell fraction using three deconvolution methods and the true fraction in the artificial mixture per cell type. **Figure S7.** Comparison of the estimated cell proportions using CP/QP using an IDOL-optimized library restricted to the Illumina HumanMethylation450K k array versus the reconstructed (true) DNA fraction in the artificial DNA mixtures arrayed in the 450 k platform. (PDF 618 kb)

Additional file 2: Gene Ontology enrichment of the probes contained in the L-DMR IDOL library. (CSV 28 kb)

Additional file 3: GSEA enrichment using the curated set 7 (immune profiles) of the probes contained in the L-DMR IDOL library. (CSV 13 kb)

Additional file 4: L-DMR IDOL library. (CSV 113 kb)

Additional file 5: L-DMR IDOL 450 K legacy library. (CSV 88 kb)

## Abbreviations

Bcells: B lymphocytes
CD4T: CD4+ T cells
CD8T: CD8+ T cells
CP/QP: Constrained projection/ quadratic programming
EWAS: Epigenome-wide association studies
FACS: Fluorescence activated cell sorting
IDOL: Identifying Optimal Libraries
L-DMR: Leukocyte differentially methylated regions
Mono: Monocytes
Neu: Neutrophils
NK: Natural killer cells
NLR: Neutrophil to lymphocyte ratio
R^2^: Coefficient of determination
RMSE: Root mean square error

## References

[1] Breton CV, Marsit CJ, Faustman E, Nadeau K, Goodrich JM, Dolinoy DC (2017). Small-magnitude effect sizes in epigenetic end points are important in children’s environmental health studies: the Children’s Environmental Health and Disease Prevention Research Center’s Epigenetics Working Group. Environ Health Perspect.

[2] Christensen BC, Houseman EA, Marsit CJ, Zheng S, Wrensch MR, Wiemels JL (2009). Aging and environmental exposures alter tissue-specific DNA methylation dependent upon CpG island context. PLoS Genet.

[3] Levenson VV (2010). DNA methylation as a universal biomarker. Expert Rev Mol Diagn.

[4] Houseman EA, Kim S, Kelsey KT, Wiencke JK (2015). DNA methylation in whole blood: uses and challenges. Curr Environ Heal Rep.

[5] Titus AJ, Gallimore RM, Salas LA, Christensen BC (2017). Cell-type deconvolution from DNA methylation: a review of recent applications. Hum Mol Genet.

[6] Teschendorff AE, Zheng SC (2017). Cell-type deconvolution in epigenome-wide association studies: a review and recommendations. Epigenomics..

[7] Houseman EA, Accomando WP, Koestler DC, Christensen BC, Marsit CJ, Nelson HH (2012). DNA methylation arrays as surrogate measures of cell mixture distribution. BMC Bioinformatics..

[8] Houseman EA, Kelsey KT, Wiencke JK, Marsit CJ (2015). Cell-composition effects in the analysis of DNA methylation array data: a mathematical perspective. BMC Bioinformatics..

[9] Zheng SC, Beck S, Jaffe AE, Koestler DC, Hansen KD, Houseman AE (2017). Correcting for cell-type heterogeneity in epigenome-wide association studies: revisiting previous analyses. Nat Methods.

[10] Guo S, Diep D, Plongthongkum N, Fung H-L, Zhang K, Zhang K (2017). Identification of methylation haplotype blocks aids in deconvolution of heterogeneous tissue samples and tumor tissue-of-origin mapping from plasma DNA. Nat Genet.

[11] Koestler DC, Usset J, Christensen BC, Marsit CJ, Karagas MR, Kelsey KT (2017). DNA methylation-derived neutrophil-to-lymphocyte ratio: an epigenetic tool to explore cancer inflammation and outcomes. Cancer Epidemiol Biomark Prev.

[12] Wiencke JK, Koestler DC, Salas LA, Wiemels JL, Roy RP, Hansen HM (2017). Immunomethylomic approach to explore the blood neutrophil lymphocyte ratio (NLR) in glioma survival. Clin Epigenetics.

[13] Reinius LE, Acevedo N, Joerink M, Pershagen G, Dahlén SE, Greco D (2012). Differential DNA methylation in purified human blood cells: Implications for cell lineage and studies on disease susceptibility. PLoS One.

[14] Teschendorff AE, Breeze CE, Zheng SC, Beck S (2017). A comparison of reference-based algorithms for correcting cell-type heterogeneity in Epigenome-Wide Association Studies. BMC Bioinformatics..

[15] Goode DK, Obier N, Vijayabaskar MS, Lie-A-Ling M, Lilly AJ, Hannah R (2016). Dynamic gene regulatory networks drive hematopoietic specification and differentiation. Dev Cell.

[16] Zhou W, Laird PW, Shen H (2017). Comprehensive characterization, annotation and innovative use of Infinium DNA methylation BeadChip probes. Nucleic Acids Res.

[17] Logue MW, Smith AK, Wolf EJ, Maniates H, Stone A, Schichman SA (2017). The correlation of methylation levels measured using Illumina 450K and EPIC BeadChips in blood samples. Epigenomics.

[18] Koestler DC, Jones MJ, Usset J, Christensen BC, Butler RA, Kobor MS (2016). Improving cell mixture deconvolution by identifying optimal DNA methylation libraries (IDOL). BMC Bioinformatics.

[19] Aryee MJ, Jaffe AE, Corrada-Bravo H, Ladd-Acosta C, Feinberg AP, Hansen KD (2014). Minfi: A flexible and comprehensive Bioconductor package for the analysis of Infinium DNA methylation microarrays. Bioinformatics.

[20] Saijo K, Schmedt C, Su I-H, Karasuyama H, Lowell CA, Reth M (2003). Essential role of Src-family protein tyrosine kinases in NF-kappaB activation during B cell development. Nat Immunol.

[21] Miceli MC, Parnes JR (1993). Role of CD4 and CD8 in T cell activation and differentiation. Adv Immunol.

[22] Addison EG, North J, Bakhsh I, Marden C, Haq S, Al-Sarraj S (2005). Ligation of CD8alpha on human natural killer cells prevents activation-induced apoptosis and enhances cytolytic activity. Immunology.

[23] Fazi F, Rosa A, Fatica A, Gelmetti V, De Marchis ML, Nervi C (2005). A minicircuitry comprised of microRNA-223 and transcription factors NFI-A and C/EBPalpha regulates human granulopoiesis. Cell.

[24] Vian L, Di Carlo M, Pelosi E, Fazi F, Santoro S, Cerio AM (2014). Transcriptional fine-tuning of microRNA-223 levels directs lineage choice of human hematopoietic progenitors. Cell Death Differ.

[25] Yang K, Shrestha S, Zeng H, Karmaus PWF, Neale G, Vogel P (2013). T cell exit from quiescence and differentiation into Th2 cells depend on Raptor-mTORC1-mediated metabolic reprogramming. Immunity.

[26] Yusuf N, Hidalgo B, Irvin MR, Sha J, Zhi D, Tiwari HK (2017). An epigenome-wide association study of inflammatory response to fenofibrate in the Genetics of Lipid Lowering Drugs and Diet Network. Pharmacogenomics.

[27] Puck A, Aigner R, Modak M, Cejka P, Blaas D, Stöckl J (2015). Expression and regulation of Schlafen (SLFN) family members in primary human monocytes, monocyte-derived dendritic cells and T cells. Results Immunol.

[28] Stehbens SJ, Paszek M, Pemble H, Ettinger A, Gierke S, Wittmann T (2014). CLASPs link focal-adhesion-associated microtubule capture to localized exocytosis and adhesion site turnover. Nat Cell Biol.

[29] de Goede OM, Lavoie PM, Robinson WP (2016). Characterizing the hypomethylated DNA methylation profile of nucleated red blood cells from cord blood. Epigenomics..

[30] de Goede OM, Razzaghian HR, Price EM, Jones MJ, Kobor MS, Robinson WP (2015). Nucleated red blood cells impact DNA methylation and expression analyses of cord blood hematopoietic cells. Clin Epigenetics.

[31] Bakulski KM, Feinberg JI, Andrews SV, Yang J, Brown S, L McKenney S (2016). DNA methylation of cord blood cell types: Applications for mixed cell birth studies. Epigenetics.

[32] Gervin K, Page CM, Aass HCD, Jansen MA, Fjeldstad HE, Andreassen BK (2016). Cell type specific DNA methylation in cord blood: a 450K-reference dataset and cell count-based validation of estimated cell type composition. Epigenetics.

[33] Shvetsov YB, Song M-A, Cai Q, Tiirikainen M, Xiang Y-B, Shu X-O (2015). Intraindividual variation and short-term temporal trend in DNA methylation of human blood. Cancer Epidemiol Biomark Prev.

[34] Urdinguio RG, Torró MI, Bayón GF, Álvarez-Pitti J, Fernández AF, Redon P (2016). Longitudinal study of DNA methylation during the first 5 years of life. J Transl Med.

[35] Tan Q, Heijmans BT, Hjelmborg JVB, Soerensen M, Christensen K, Christiansen L (2016). Epigenetic drift in the aging genome: a ten-year follow-up in an elderly twin cohort. Int J Epidemiol.

[36] Kananen L, Marttila S, Nevalainen T, Kummola L, Junttila I, Mononen N (2016). The trajectory of the blood DNA methylome ageing rate is largely set before adulthood: evidence from two longitudinal studies. Age (Dordr). Age.

[37] Salas LA, Koestler DC, Butler RA, Hansen HM, Wiencke JK, Kelsey KT, et al. FlowSorted.Blood.EPIC. Bioconductor. 2018. https://bioconductor.org/packages/FlowSorted.Blood.EPIC, 10.18129/B9.bioc.FlowSorted.Blood.EPIC. Accessed 4 May 2018.

[38] Salas LA, Koestler DC, Butler RA, Hansen HM, Wiencke JK, Kelsey KT, et al. GSE110554: FlowSorted.Blood.EPIC: An optimized library for reference-based deconvolution of whole-blood biospecimens assayed using the Illumina HumanMethylationEPIC BeadArray (II). Gene Expression Omnibus. 2018. https://www.ncbi.nlm.nih.gov/geo/query/acc.cgi?acc=GSE110554. [cited 2018 May 4]. 10.1186/s13059-018-1448-7 PMC5975716 29843789

[39] Xu Z, Niu L, Li L, Taylor JA (2016). ENmix: a novel background correction method for Illumina HumanMethylation450 BeadChip. Nucleic Acids Res.

[40] R Core Team (2017). R: a language and environment for statistical computing.

[41] Fortin J-P, Triche TJ, Hansen KD (2017). Preprocessing, normalization and integration of the Illumina HumanMethylationEPIC array with minfi. Bioinformatics.

[42] Touleimat N, Tost J (2012). Complete pipeline for Infinium(®) Human Methylation 450K BeadChip data processing using subset quantile normalization for accurate DNA methylation estimation. Epigenomics.

[43] Salas LA, Koestler DC, Butler RA, Hansen HM, Wiencke JK, Kelsey KT, et al. GSE110530: Longitudinal dataset: An optimized library for reference-based deconvolution of whole-blood biospecimens assayed using the Illumina HumanMethylationEPIC BeadArray (I). Gene Expression Omnibus. 2018. https://www.ncbi.nlm.nih.gov/geo/query/acc.cgi?acc=GSE110530. Accessed 4 May 2018. 10.1186/s13059-018-1448-7 PMC5975716 29843789

[44] Koestler DC, Christensen BC, Wiencke JK, Kelsey KT. GSE77797: DNA methylation profiling of whole blood and reconstructed mixtures of purified leukocytes isolated from human adult blood. Gene Expression Omnibus. 2016. https://www.ncbi.nlm.nih.gov/geo/query/acc.cgi?acc=GSE77797. Accessed 4 May 2018.

[45] Salas LA, Koestler DC, Butler RA, Hansen HM, Wiencke JK, Kelsey KT, et al. GSE112618: FACS validation dataset: An optimized library for reference-based deconvolution of whole-blood biospecimens assayed using the Illumina HumanMethylationEPIC BeadArray (III). Gene Expression Omnibus. 2018. https://www.ncbi.nlm.nih.gov/geo/query/acc.cgi?acc=GSE112618. Accessed 4 May 2018. 10.1186/s13059-018-1448-7 PMC5975716 29843789

[46] Accomando WP, Wiencke JK, Houseman EA, Nelson HH, Kelsey KT (2014). Quantitative reconstruction of leukocyte subsets using DNA methylation. Genome Biol.

[47] Phipson B, Maksimovic J, Oshlack A (2016). missMethyl: an R package for analyzing data from Illumina’s HumanMethylation450 platform. Bioinformatics.

[48] Salas LA, Koestler DC, Butler RA, Hansen HM, Wiencke JK, Kelsey KT, et al. GSE110555: SuperSeries: an optimized library for reference-based deconvolution of whole-blood biospecimens assayed using the Illumina HumanMethylationEPIC BeadArray. Gene Expression Omnibus. 2018. https://www.ncbi.nlm.nih.gov/geo/query/acc.cgi?acc=GSE110555. Accessed 4 May 2018. 10.1186/s13059-018-1448-7 PMC5975716 29843789

[49] Salas LA, Koestler DC, Butler RA, Hansen HM, Wiencke JK, Kelsey KT, et al. FlowSorted.Blood.EPIC. GitHub. 2018. https://github.com/immunomethylomics/FlowSorted.Blood.EPIC. Accessed 4 May 2018.

[50] Salas LA, Koestler DC, Butler RA, Hansen HM, Wiencke JK, Kelsey KT, et al. Immunomethylomics/FlowSorted.Blood.EPIC: FlowSorted.Blood.EPIC v.0.99.36. Zenodo. 2018. 10.5281/ZENODO.1241200. Accessed 4 May 2018.

[51] Salas LA. v.1.0 immunomethylomics/Analysis_FlowSorted.Blood.EPIC: analysis scripts. 2018. 10.5281/zenodo.1243840. Accessed 4 May 2018.

