# Supplementary material for: An optimized library for reference-based deconvolution of whole-blood biospecimens assayed using the Illumina HumanMethylationEPIC BeadArray
Source: Genome Biol. 2018 May 29;19:64. doi: 10.1186/s13059-018-1448-7 (PMC5975716; doi:10.1186/s13059-018-1448-7)

Additional file 1: Fig. S1 to Fig. S7 and Table S1

An optimized library for reference-based deconvolution of whole-blood biospecimens assayed using the Illumina HumanMethylationEPIC BeadArray

Authors: Lucas A. Salas[1†], Devin C. Koestler[2†], Rondi Butler[3], Helen Hansen[4], John K. Wiencke[4], Karl T. Kelsey[3], Brock C. Christensen[1,5*]

Affiliations:

[1]Department of Epidemiology, Geisel School of Medicine, Dartmouth College, Lebanon, NH.

[2] Department of Biostatistics, University of Kansas Medical Center, Kansas City, KS.

[3] Departments of Epidemiology and Pathology and Laboratory Medicine, Brown University, Providence, RI.

[4] Department of Neurological Surgery, Institute for Human Genetics, University of California San Francisco, San Francisco CA.

[5]Departments of Molecular and Systems Biology, and Community and Family Medicine, Geisel School of Medicine, Dartmouth College, Lebanon NH.

[*]Correspondence to: Brock.C.Christensen@dartmouth.edu

[†]Equal contributors.

Table of Contents

Fig. S1. Estimated cell purity by flow cytometry per cell type........ 2

Fig. S2. Heatmap based on a hierarchical cluster of purified cell types and cell mixtures based on the array SNPs. 3

Fig. S3. Association between the top 20 principal components and potential confounders for DNA methylation. 4

Fig. S4. Iterative testing of different L-DMR library sizes using the IDOL optimization algorithm. 5

Table S1. Cell composition percentages for the artificial reconstruction samples. 6

Fig. S5. Comparison of several probe selection methods and estimate cell proportions using constrained projection/ quadratic programming CP/QP versus the reconstructed (true) DNA fraction in the artificial DNA mixtures 7

Fig. S6. Bland-Altman plots comparing the mean differences between the estimated cell fraction using three deconvolution methods and the true fraction in the artificial mixture per cell type...... 8

Fig. S7. Comparison of the estimate cell proportions using constrained projection/ quadratic programming CP/QP using an IDOL optimized library restricted to the Illumina HumanMethylation 450k-450k array versus the reconstructed (true) DNA fraction in the artificial DNA mixtures arrayed in the 450k platform 9

Fig. S1. Estimated cell purity by flow cytometry per cell type

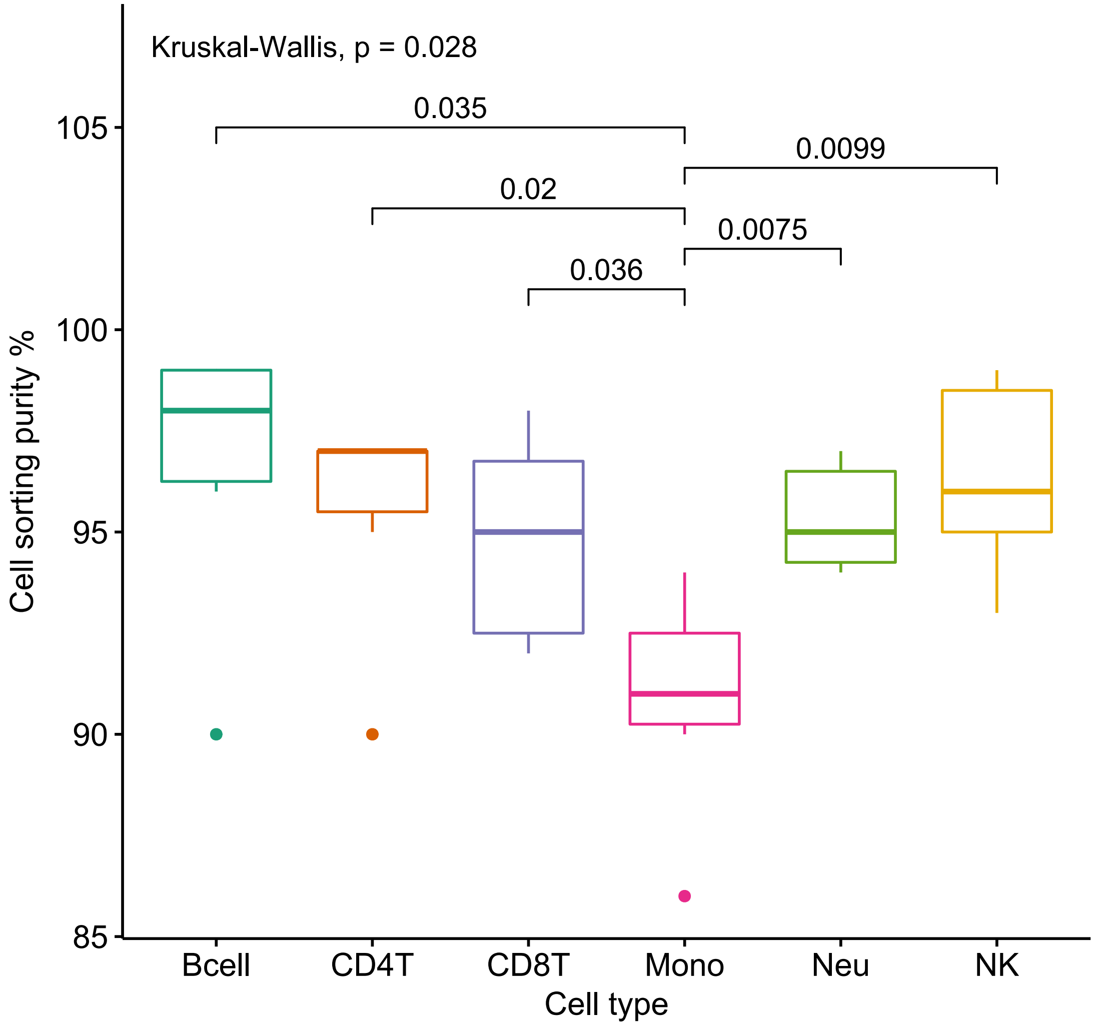

Monocyte estimates purity were statistically significant different to the other cell types.

Fig. S2. Heatmap based on a hierarchical cluster of purified cell types and cell mixtures based on the array SNPs.

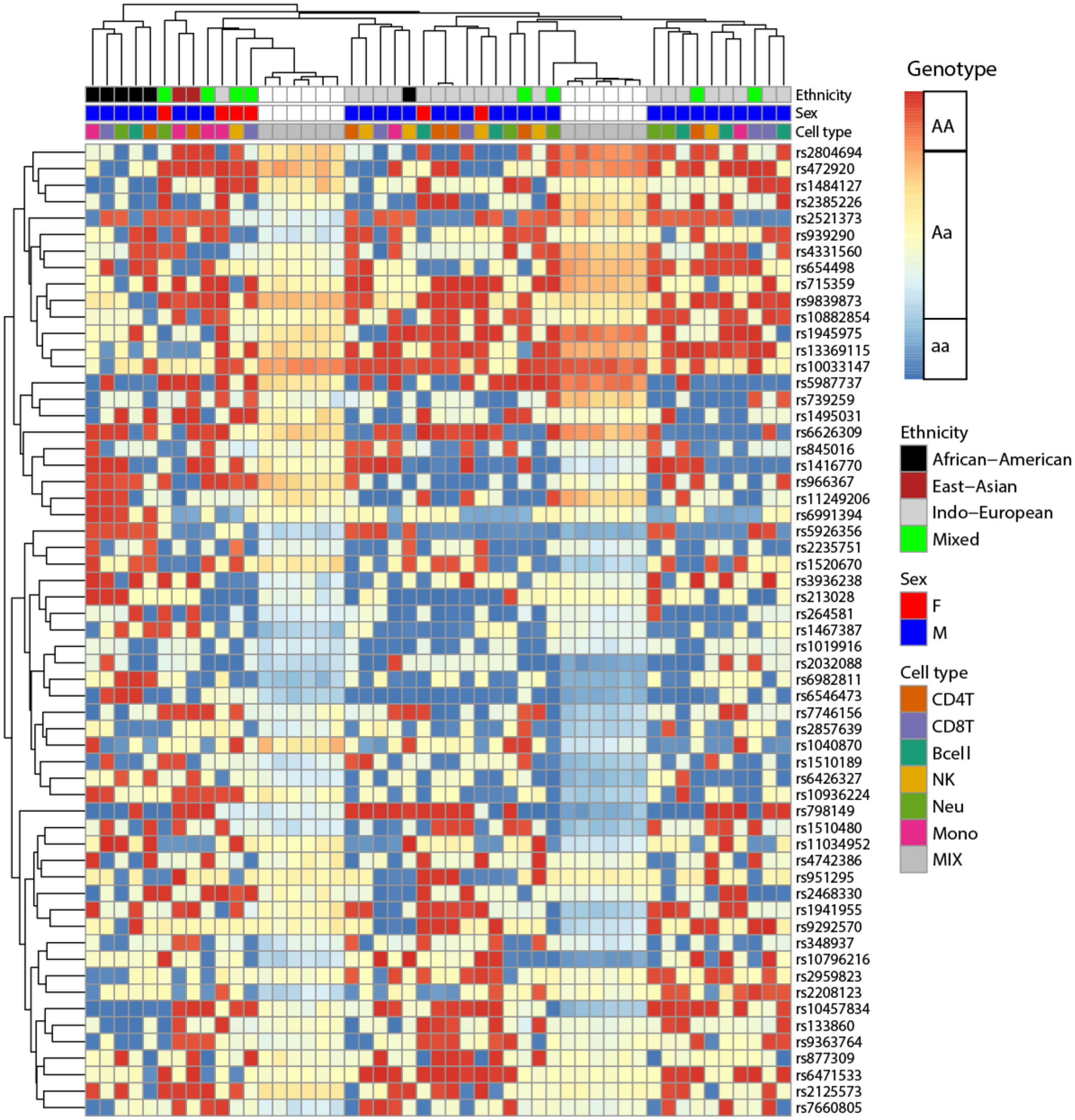

Notes: 1) Most of the African American and East-Asian clustered according to the 59 control SNPs in the array. Ethnic groups correspond to a very broad categorization, for specific self-attributed ethnicity background see the database information. 2) Although the genotype scale is based on β-values, the heatmap should be interpreted as three genotype categories: red (>0.8) AA two major alleles, yellow (>0.2-≤0.8) Aa or one minor allele, and blue (≤0.2) aa or two minor alleles present on the SNP.

Fig. S3. Association between the top 20 principal components and potential confounders for DNA methylation.

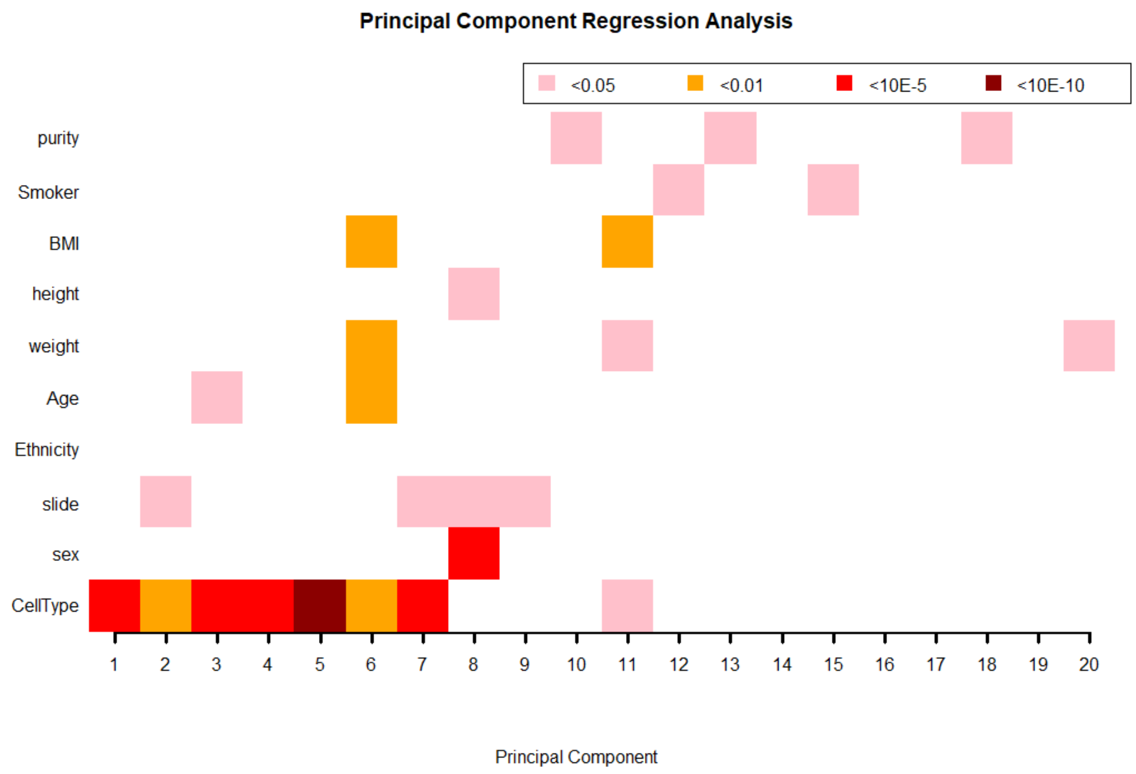

Abbreviations: purity: flow sorted cell purity estimations, smoker: current vs never smoker, BMI: body mass index, age: age in years, ethnicity: African-American, East-Asian, Indo-European, Mixed (Hispanic, others).

Fig. S4. Iterative testing of different L-DMR library sizes using the IDOL optimization algorithm.

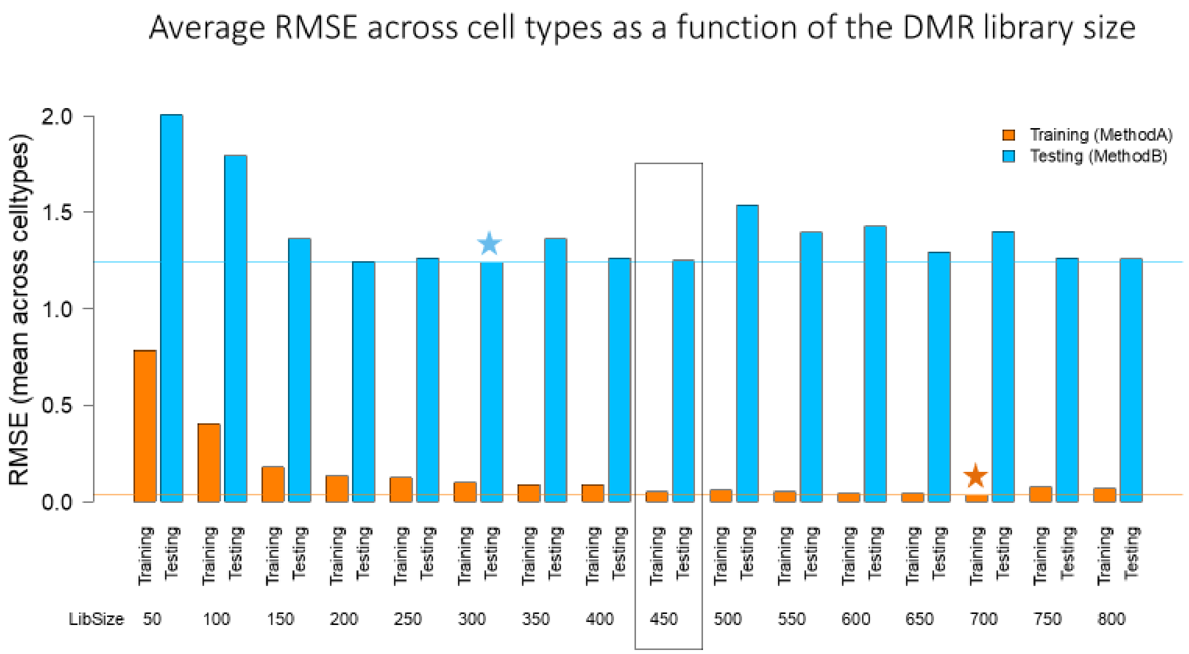

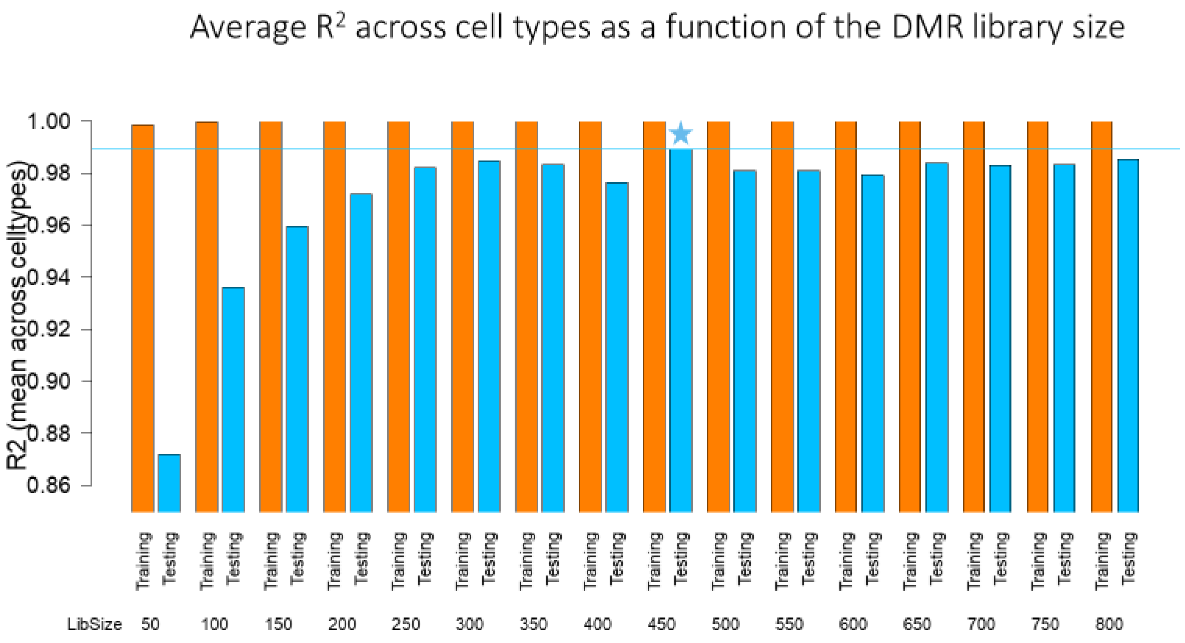

Table S1. Cell composition percentages for the artificial reconstruction samples.

Mixture Reconstruction	Sample	Cell Percentage (%)					
		Lymphoid Lineage				Myeloid Lineage	
		CD4T	CD8T	Bcell	NK	Mono	Neu
Method a	1a	13	11	16	12	23	25
	2a	7	19	19	15	19	21
	3a	6	33	8	11	19	23
	4a	16	29	7	15	22	11
	5a	11	20	20	22	10	17
	6a	18	13	26	15	22	6
Method b	1b	13	2	1	4	5	75
	2b	16	11	1	2	7	63
	3b	9	6	2	0	10	73
	4b	14	8	2	3	6	67
	5b	12	5	6	7	4	66
	6b	15	4	4	2	5	70

Fig. S5. Comparison of several probe selection methods and estimate cell proportions using constrained projection/ quadratic programming CP/QP versus the reconstructed (true) DNA fraction in the artificial DNA mixtures

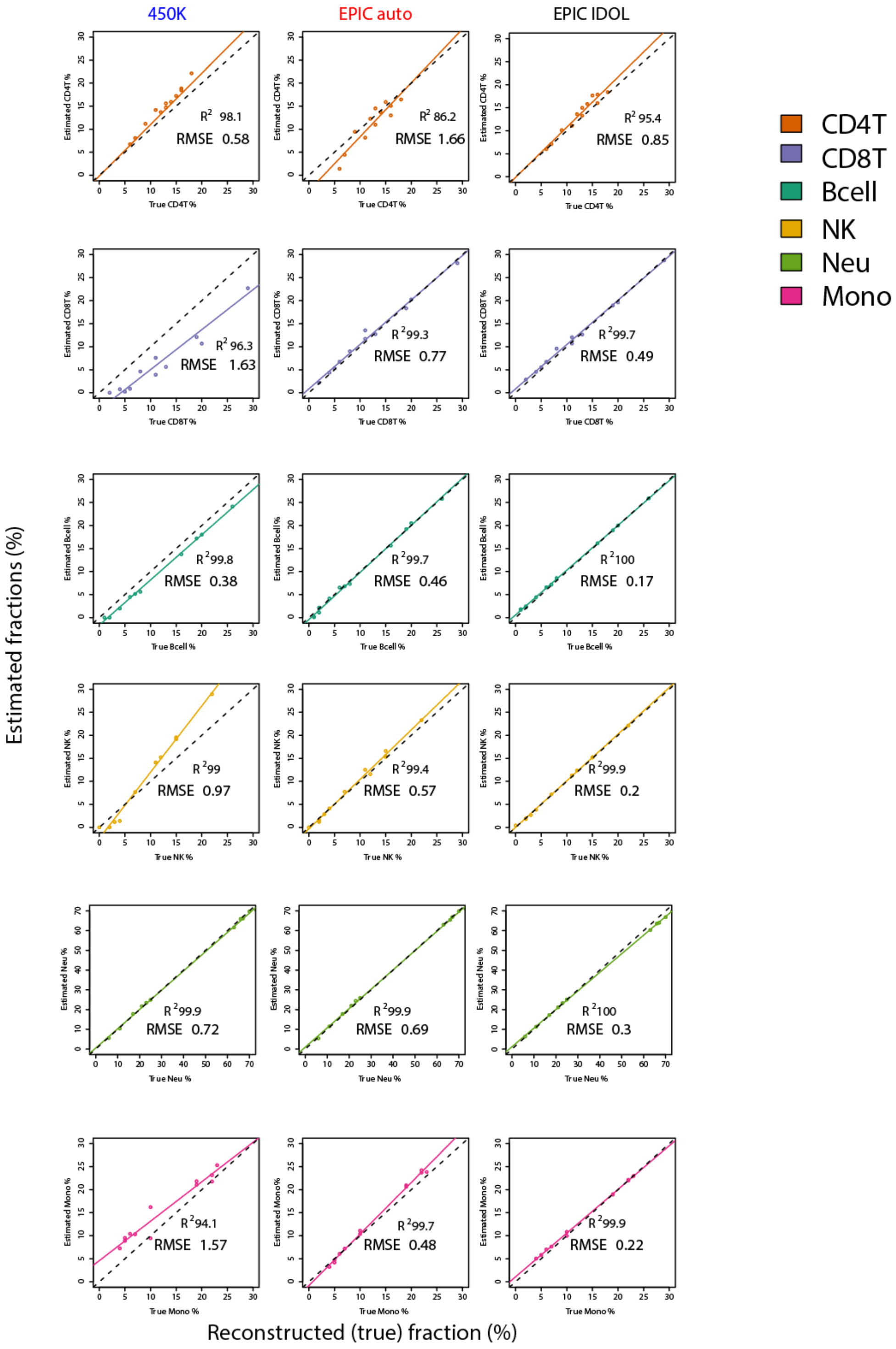

Fig. S6. Bland-Altman plots comparing the mean differences between the estimated cell fraction using three deconvolution methods and the true fraction in the artificial mixture per cell type

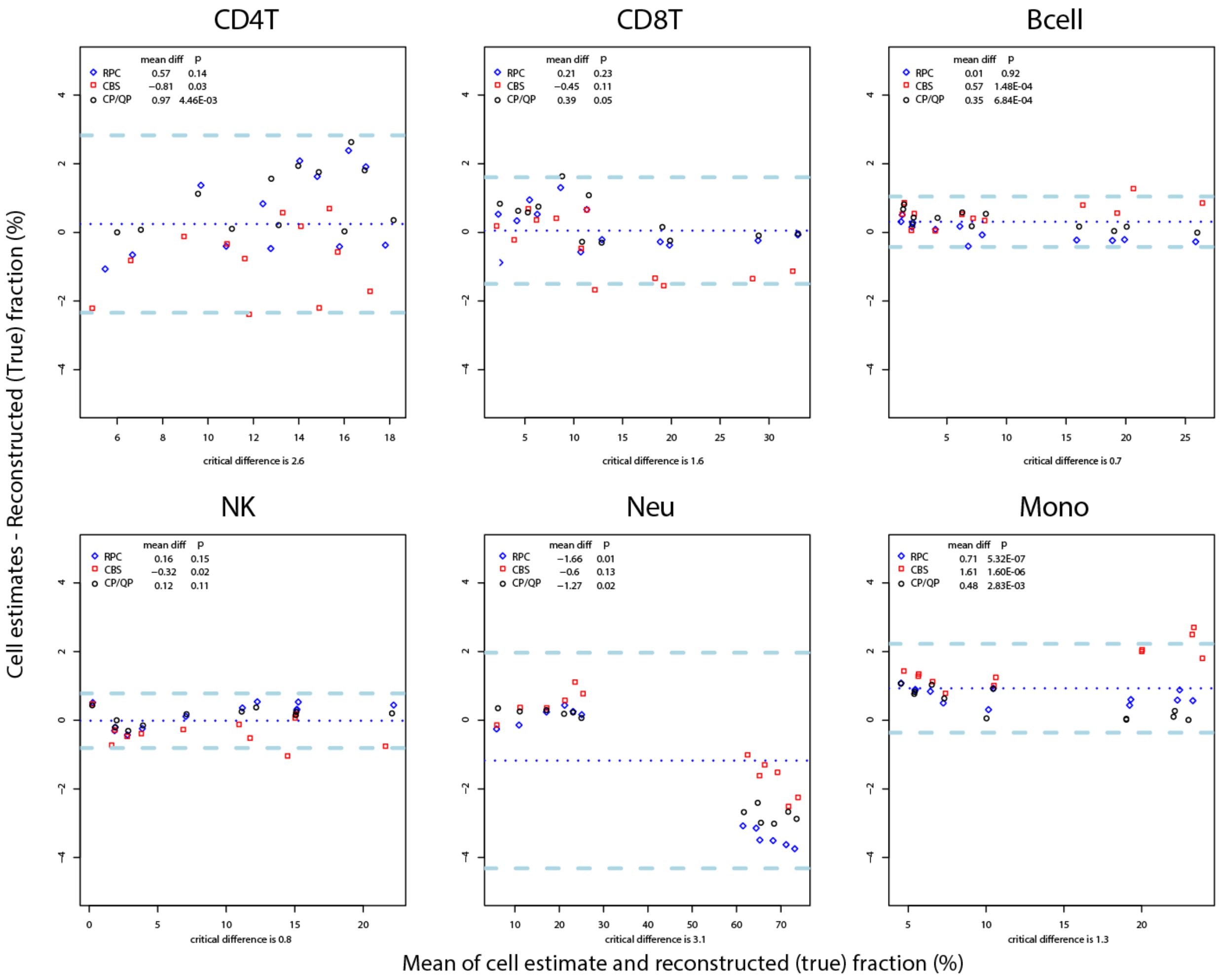

Notes: in the Bland Altman plots, the x axes show the mean of each estimate versus the corresponding true value, the y axes show the mean difference for Robust Partial Correlation-RPC (blue diamonds), CIBERSORT-CBS (red squares), and constrained projection/quadratic programming-CP/QP (black circles). The confidence bands (dashed horizontal lines) are 2 SD (critical 95% confidence interval difference) from the mean of the differences (dark dotted line). The mean difference per method and the paired t-test p-value are summarized on the top left of each plot.

Fig. S7. Comparison of the estimate cell proportions using constrained projection/ quadratic programming CP/QP using an IDOL optimized library restricted to the Illumina HumanMethylation 450k-450k array versus the reconstructed (true) DNA fraction in the artificial DNA mixtures arrayed in the 450k platform

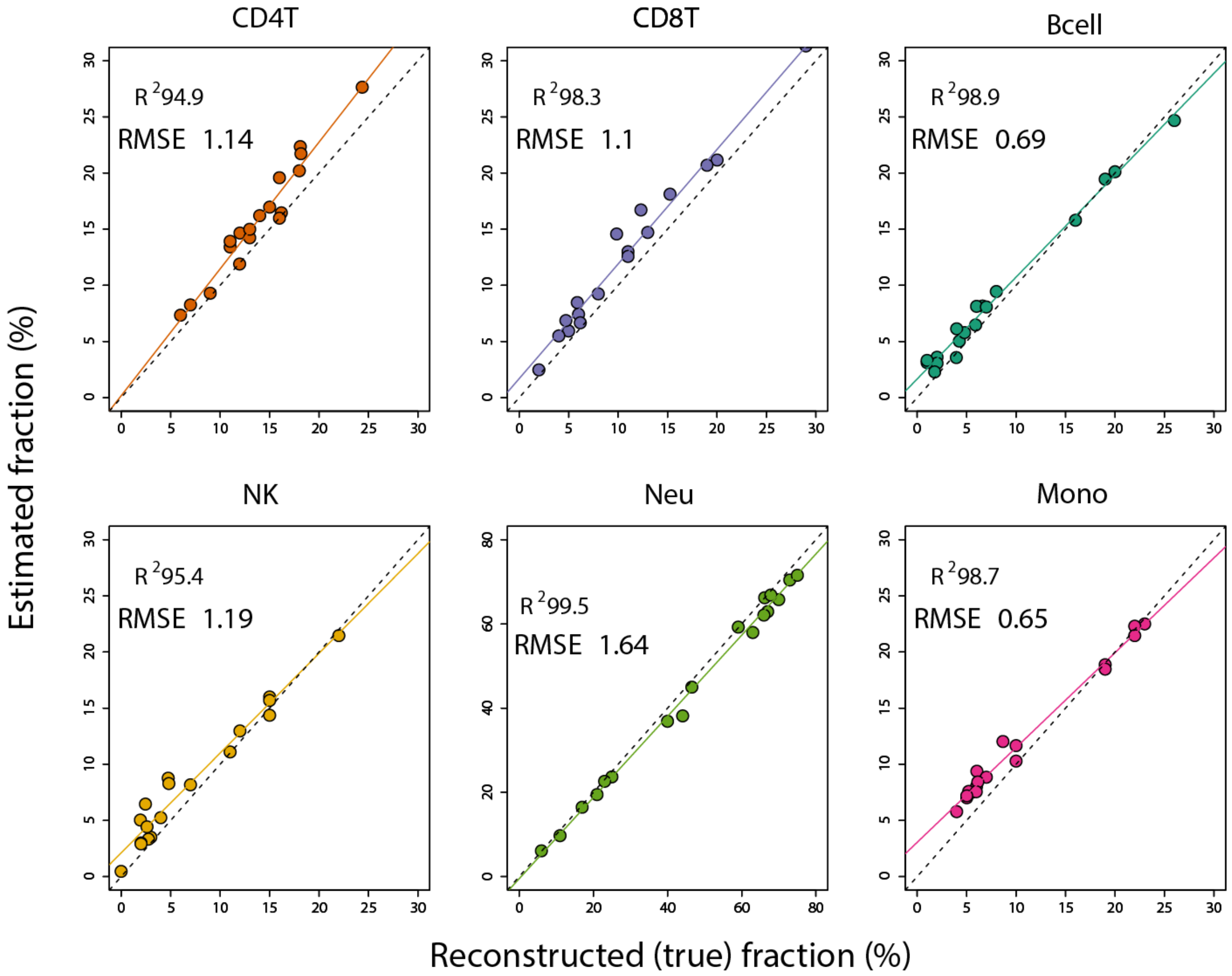

Supplement: Supplementary file 1 — Figure S1. Estimated cell purity by flow cytometry per cell type. Figure S2. Heatmap based on a hierarchical cluster of purified cell types and cell mixtures based on the array SNPs. Figure S3. Association between the top 20 principal components and potential confounders for DNA methylation. Figure S4. Iterative testing of different L-DMR library sizes using the IDOL optimization algorithm. Table S1. Cell composition percentages for the artificial reconstruction samples. Figure S5. Comparison of several probe selection methods and estimated cell proportions using constrained projection/quadratic programming (CP/QP) versus the reconstructed (true) DNA fraction in the artificial DNA mixtures. Figure S6. Bland-Altman plots comparing the mean differences between the estimated cell fraction using three deconvolution methods and the true fraction in the artificial mixture per cell type. Figure S7. Comparison of the estimated cell proportions using CP/QP using an IDOL-optimized library restricted to the Illumina HumanMethylation450K k array versus the reconstructed (true) DNA fraction in the artificial DNA mixtures arrayed in the 450 k platform. (PDF 618 kb) [file 13059_2018_1448_MOESM1_ESM.pdf]
